# Occurrence and antimicrobial susceptibility of enteric rods and pseudomonads isolated from the dental prostheses biofilm

**DOI:** 10.1590/1678-775720160032

**Published:** 2016

**Authors:** Sanrrangers Sales Silva, Maximilo de Oliveira Ribeiro, Francisco Isaac Fernandes Gomes, Hellíada Vasconcelos Chaves, Antonio Alfredo Rodrigues e Silva, Iriana Carla Junqueira Zanin, Francisco Cesar Barroso Barbosa

**Affiliations:** 1 Universidade Federal do Ceará, Faculdade de Odontologia, Campus Sobral, Sobral, CE, Brasil; 2 Universidade Federal do Ceará, Faculdade de Medicina, Campus Sobral,Sobral, CE, Brasil; 3 Agência Nacional de Vigilância Sanitária, Brasília, DF, Brasil

**Keywords:** Biofilms, Enterobacteriaceae, Pseudomonas, Drug resistance

## Abstract

Aspiration of oral bacteria leads to cardiac and respiratory infectious diseases and dentures can act as a reservoir for pathogenic microorganisms.

**Objective:**

To determine the occurrence and the *in vitro* antimicrobial susceptibility of enteric rods and pseudomonads from the denture biofilm of 52 subjects at the Center for Dental Specialties of Sobral/ Ceara, Brazil.

**Material and Methods:**

Denture biofilm was collected and samples plated on MacConkey agar. The isolated bacterial colonies were identified using the BBL Crystal enteric/non-fermenter system. Antibiotic bacterial susceptibility was assessed by the disc diffusion method of amoxicillin, amoxicillin/clavulanic acid, doxycycline, tetracycline, tobramycin, imipenem, cefotaxime, and ciprofloxacin. The Minimum Inhibitory Concentration (MIC) of cefotaxime, tobramycin, doxycycline, imipenem, and ciprofloxacin was determined for 40 species by E-test.

**Results:**

34 subjects (65.4%) harbored enteric rods in their prostheses. *Klebsiella pneumoniae* (26.5%), *Escherichia coli* (23.5%), and *Enterobacter aerogenes* (23.5%) were the most prevalent species. All organisms were susceptible to ciprofloxacin and most species were resistant to amoxicillin or amoxicillin/clavulanic acid, demonstrating variable sensitivity patterns to other antimicrobials. However, the MIC showed the emergence of strains with reduced sensitivity to ciprofloxacin (MIC_90_≥3 μg/ mL) and cefotaxime (MIC_90_≥2 μg/mL).

**Conclusion:**

The findings show high prevalence of nosocomial diseases-related bacterial species and low susceptibility to antimicrobial drugs. Therefore, these results imply caution against the indiscriminate use of broad spectrum antibiotics in dental practice.

## INTRODUCTION

Variations in the oral microbiota are directly related to age and have been attributed to the use of dentures^16^. The oral health status declines as a result of the aging process and individuals culminate with the need of dental prostheses, affecting their health, functional activities, and self-esteem^8^. Worldwide, 810 million people are aged 60 or over, which is predicted to increase to at least two billion by 2050, 22% of the entire global population, and this demographic change will result in significant challenges for oral health care delivery, to an increasingly aged population with declining oral health^13,19,31^.

These individuals may not be able to clean their dentures properly, which in turn could result in the formation of a potentially pathogenic biofilm, exposing these individuals to the possibility of systemic severe infections. Currently, denture liners are available as silicone-based and acrylic resin-based. The adhesion to these materials depends on the properties of the surface of the microbial cells that will adhere and form biofilm, thus forming a complex three-dimensional architecture. One of the problems directly related to these materials is still the accumulation of biofilm on dentures, which in turn has received little attention by patients and general clinicians as its dental counterpart^32^.

The presence of enteric rods and pseudomonads in the denture biofilm should be highlighted because of their pathogenic potential and ability to adhere to solid surfaces^2,17^. In addition, infections caused by these organisms can be difficult to treat as a result of the bacterial resistance to a variety of antibiotics, including p-lactams, amoxicillin/clavulanic acid, cephalosporins, aminoglycosides, carbapenems, chloramphenicol, aztreonam, trimethoprim/ sulfametaxazol, tetracycline, and doxycycline^5,24^.

The clinical importance of the non-fermenters gram-negative bacilli presence in the denture biofilm has significantly increased because of infections, high rates of morbidity, mortality in hospitalized subjects, and high levels of resistance. The dissemination of gram-negative bacteria with acquired antimicrobial drugs resistance is becoming a global problem. The propagation and dissemination of these microorganisms have already been confirmed^11,25^.

Enteric microorganisms and pseudomonads have demonstrated significant levels of resistance for all β-lactams, except for imipenem and meropenem. However, there are current reports of resistant enteric rods to carbapenems^30^. Among bacterial isolates resistant to ampicillin or amoxicillin, the most resistant were β-lactamase producers. The production of these hydrolytic enzymes seems to be the major mechanism of resistance to β-lactams, excluding most pseudomonads, in which β-lactamases were not detected. On the other hand, resistance to tetracycline was widely disseminated in the microbial enteric rods, and many of tested microorganisms were resistant^25^.

Having known such information, our hypotheses are that wearing dental prostheses older people may harbor superinfecting microorganisms in their oral cavity as a result of the biofilm formation on the surface of these appliances as well as these bacterial species could possess antimicrobial resistance. Therefore, we consider relevant to determine the prevalence of *Enterobacteriaceae* and *Pseudomonadaceae* species isolated from the denture biofilm and to assess their *in vitro* susceptibility to antimicrobial drugs, since these opportunistic microorganisms were previously reported to be on the dental prostheses biofilm^15,28,29^.

## MATERIAL AND METHODS

### Study population

Enteric microorganisms were isolated from the inner surface of dentures of 52 subjects within a 2-year follow-up period (2007-2008) at the Center for Dental Specialties - Sobral, State of Ceará, Brazil. The requirements for inclusion in this study were: no history of diabetes, no use of antimicrobials in the past three months, nor other medication that could affect their systemic or local immune system. All subjects signed a written consent form that was approved by the Institutional Review Board of Federal University of Ceará (COMEPE n° 258/07).

### Data collection

All subjects answered a questionnaire about their systemic health and a dental and soft tissue examination was performed to assess their oral conditions. The resulting findings were recorded in the medical record of each patient. Initially, the surfaces of the dentures were thoroughly dried with sterilized gauze to avoid contamination by saliva. The biofilm on the inner surface of complete dentures was collected with the aid of a sterile swab, and the samples were immediately placed in sterile phosphate buffered saline (PBS, pH 7.5, 0.8% NaCl) to the Department of Microbiology and Parasitology, Federal University of Ceará, in Sobral, and processed within a maximum of 2 hours after collection. Samples were dispersed by agitation (30 seconds) and serially diluted (10^−1^ and 10^−2^) in PBS. Aliquots of 100 μL of the solutions obtained were plated on MacConkey agar (Acumedia, Lansing, Michigan, USA). Then, the plates were incubated at 36°C±1°C for 24 hours.

### Microorganisms and microbial identification

The isolated bacterial colonies were identified by Gram staining, colony morphology on MacConkey agar plates (Acumedia, Lansing, Michigan, USA), production of oxidase (Oxidase Newprov Strips, Pinhais, SP, Brazil), and biochemical identification kits (Kit for Identification of *Enterobacteriaceae* Newprov). The definitive identification of glucose enteric bacilli fermenters, oxidase-negative bacilli, and non-fermenter bacilli was carried out by the system BBL Crystal Enteric/Nonfermenter (Becton Dickinson Systems, Cockeysville, Maryland, USA) according to the manufacturer's instructions. The sample *Escherichia coli* ATCC 25922 was used as control.

### Antimicrobial susceptibility test

The identified and isolated bacterial colonies previously collected were stored in broth heart infusion medium (Acumedia, Lansing, Michigan, USA) with glycerol (3:1) at – 80°C before performing the antimicrobial susceptibility tests. Then, each strain was seeded in broth heart infusion medium (Acumedia, Lansing, Michigan, USA) and incubated in a microbiological greenhouse at 36°C±1°C for 24 hours. Thereafter, in order to perform the sensitivity test, it was used the disk diffusion method of the Clinical & Laboratory Standards Institute manual^6^. The antimicrobial drugs tested were amoxicillin (10 μg), amoxicillin/clavulanate (20/10 μg), doxycycline (30 μg), tetracycline (30 μg), tobramycin (10 μg), imipenem (10 μg), cefotaxime (30 μg), and ciprofloxacin (5 μg). Mueller Hinton agar was the medium used for this test. After seeding and incubating the samples in a microbiological greenhouse at 36°C±1°C for 24 hours, the zones of inhibition were measured and the organisms were classified as sensitive, intermediate, or resistant according to CLSI references^6,7^. A total of 52 strains of enteric rods and pseudomonads were submitted to susceptibility tests. For multidrug resistant species, the Minimum Inhibitory Concentration (MIC) was determined for the following drugs: cefotaxime (CT), tobramycin (TM), doxycycline (DC), imipenem (IP), and ciprofloxacin (CI); the methodology used was E-test® (AB Biodisk, Solna, Sweden) and interpretations were made according to the CLSI references^6,7^.

### Statistical analysis

The Mann-Whitney test was used to determine differences regarding age groups for men and women, and the chi-square test was used to access differences between genders, age groups, and time of prostheses use correlated between the presence and absence of the studied microorganisms. Differences of p<0.05 were considered statistically significant.

## RESULTS

The population consisted of 52 individuals, of whom 42 (80.8%) were women, ranging from 35 to 81 years old (61.7±10.6), and 10 (19.2%) were men, ranging from 53 to 93 years old (72±13.2). There was no statistically significant difference between men and women regarding age groups (p=0.245) and the average time of denture usage was around 11.09 years (±9.6) (Table 1). Regarding their systemic health status, 31 (59.6%) subjects reported to have systemic hypertension, five (9.6%) reported to be diagnosed with type 2 diabetes, three (5.7%) reported to have gastritis, one (1.9%) reported to be asthmatic, and one (1.9%) to have osteoporosis, while 11 (21.1%) were systemically healthy at anamnesis. Regarding their oral condition, we diagnosed three (5.7%) subjects with candidiasis, two (3.8%) with stomatitis, one (1.9%) with xerostomia, while 46 (88.4%) were found to be orally healthy at anamnesis.

**Table 1:** Age and gender distribution of subjects in the 2007-2008 period

AGE	MEN		WOMEN		TOTAL	
	n	%	n	%	n	%
35-49	0	00.0	4	9.5	4	9.5
50-64	3	30.0	19	45.2	22	42.3
65-79	5	50.0	17	40.5	22	42.3
>80	2	20.0	2	4.8	4	7.7
Total	10	19.2	42	80.5	52	100

According to Tables 2 and 3, 34 (65.4%) subjects harbored enteric bacilli and/or pseudomonas species on dental prostheses. Most of these subjects (91.2%) aged over 50. Also, the studied microorganisms were found in 15 (75%) subjects in the group of subjects who wore dental prostheses over a 10-year period of time (Table 4). However, there was no statistically significant difference between the time of prostheses usage and the presence or absence of microorganisms (p>0.05).

**Table 2:** Bacterial species distribution isolated from dental prostheses biofilm

MICROORGANISM	MEN		WOMEN	
	n	%	n	%
Only *enteric bacilli*	6	60.0	24	57.1
Only *Pseudomonadaceae*	0	00.0	1	2.4
*Enteric bacilli* and *Pseudomonadaceae*	0	00.0	1	2.4
Other species of microrganisms	0	00.0	2	4.8
Absence of microrganisms	4	40.0	14	33.3
Total of individuals	10	100.0	42	100.0

**Table 3:** Number of analyzed subjects with dental prostheses harboring enteric rods, *Pseudomonas, Acinetobacter* spp., and *Burkholderia* spp. associated with groups of age and gender

AGE	MEN		WOMEN		TOTAL	
	h/n	h%	h/n	h%	h/n	h%
35-49	0/0	0.0	3/4	75.0	3/4	75.0
50-64	2/3	66.7	11/19	57.9	13/22	59.1
65-79	3/5	60.0	12/17	70.6	15/22	68.2
≥80	1/2	50.0	2/2	100.0	3/4	75.0
All ages	6/10	60.0	28/42	66.7	34/52	65.4

n= subjects in each group

h= harboring subjects in each group

h%= percentage of harboring subjects

**Table 4:** Number of subjects wearing harbored or non-harbored dental prostheses by enteric rods, *Pseudomonas, Acinetobacter* spp., and *Burkholderia* spp. associated for time of dental prostheses usage

TIME OF USAGE (YEARS)	CONTAMINATED		CONTAMINATED	
	h/n	h%	nh/ n	nh%
0-10	19/32	59.4	13/32	40.6
11-20	11/13	84.6	2/13	15.4
21-30	4/6	66.6	2/6	33.4
31-40	0/1	0.0	1/1	100.0

n= subjects in each group

h= harboring subjects in each group

h%= percentage of harboring subjects

nh= non-harboring subjects in each group

nh%= percentage of non-harboring subjects

Gender did not influence the colonization of the prosthesis, since no significant difference was observed in this study (p=0.74). Across the 42-woman group, 24 (57.1%) had their dentures solely contaminated with enteric bacilli, one participant (2.4%) had *Pseudomonas* species, and another one (2.4%) harbored enteric bacilli and pseudomonads. Furthermore, an 81-year old participant, who wore prostheses for 1 year, and a 59-year old subject, exceeding 20 years of denture use, had *Acinetobacter iwoffi* and *Burkholderia pseudomallei* detected on the biofilm from their dental prostheses.

Regarding the species found, *Klebsiella pneumoniae, Escherichia coli,* and *Enterobacter aerogenes* were the bacteria most prevalent detected, being isolated from dentures of nine (26.5%), eight (23.5%), and eight (23.5%) individuals, respectively. It was also detected *Citrobacter freundii* (11.8%), *Klebsiella ozaenae* (11.8%), *Klebsiella oxytoca* (8.8%), *Enterobacter cloacae* (8.8%), *Serratia marcescens* (8.8%), *Serratia liquefaciens* (8.8%), *Enterobacter gergoviae* (5.9%), *Pseudomonas aeruginosa* (5.9%), *Pseudomonas putida* (2.9%), *Enterobacter sakasakii* (2.9%), *Burkholderia pseudomallei* (2.9%), and *Acinetobacter iwoffi* (2.9%).

Table 5 lists the antimicrobial susceptibility of all detected bacterial species. All strains were susceptible to ciprofloxacin and imipenem, except one of *E. coli.* Most species were resistant to amoxicillin or amoxicillin associated with clavulanic acid. The results from the MIC test on the bacterial species showed multidrug resistance patterns (Table 6): 33.4% of the *K. pneumoniae* species were resistant to cefotaxime (CT) and 22.2% to doxycycline (DC), being 11.1% of these organisms resistant to the other analyzed antimicrobial drugs.

**Table 5:** Antimicrobial susceptibility of microorganisms isolated from denture biofilms

Microorganism	CIP	IPM	TET	CTX	AMO	DOX	AMC	TOB
*Klebsiella pneumoniae* (9)	S(9)	S(9)	S(3)I(1) R(5)	S(7)I(1) R(1)	R(9)	S(4)I(3) R(2)	S(4)I(1) R(4)	S(8)R(1)
*Escherichia coli* (8)	S(8)	S(7)R(1)	S(5)R(3)	S(6)R(2)	S(3)R(5)	S(6)I(1) R(1)	S(3)I(1) R(4)	S(7)R(1)
*Enterobacter aerogenes* (8)	S(8)	S(8)	S(6)R(2)	S(7)I(1)	R(8)	S(6)R(2)	R(8)	S(8)
*Citrobacter freundii* (4)	S(4)	S(4)	S(3)R(1)	S(2)I(1) R(1)	I(1)R(3)	S(2)I(1) R(1)	S(1)I(2) R(1)	S(4)
*Klebsiella ozaenae* (4)	S(4)	S(4)	S(4)	S(4)	S(1)R(3)	S(4)	S(4)	S(4)
*Klebsiella oxytoca* (3)	S(3)	S(3)	S(3)	S(2)R(1)	R(3)	S(3)	S(2)R(1)	S(3)
*Enterobacter cloacae* (3)	S(3)	S(3)	I(1)R(2)	S(1)I(1) R(1)	R(3)	S(1)R(2)	I(1)R(2)	S(2)R(1)
*Serratia marcescens* (3)	S(3)	S(3)	S(2)R(1)	S(2)I(1)	S(1)R(2)	S(3)	S(1)R(2)	S(2)R(1)
*Serratia liquefaciens* (2)	S(2)	S(2)	I(2)	I(1)R(1)	R(2)	S(1)R(1)	R(2)	S(2)
*Enterobacter gergoviae* (2)	S(2)	S(2)	S(1)I(1)	S(1)I(1)	R(2)	S(1)R(1)	R(2)	S(2)
*Pseudomonas aeruginosa* (2)	S(2)	S(2)	I(1)R(1)	S(1)I(1)	R(2)	R(2)	R(2)	S(2)
*Pseudomonas putida* (1)	S(1)	S(1)	I(1)	I(1)	R(1)	R(1)	R(1)	S(1)
*Enterobacter sakasakii* (1)	S(1)	S(1)	S(1)	S(1)	R(1)	S(1)	R(1)	S(1)
*Burkholderia pseudomallei* (1)	S(1)	S(1)	R(1)	I(1)	R(1)	R(1)	R(1)	S(1)
*Acinetobacter iwoffi (1)*	S(1)	S(1)	S(1)	S(1)	S(1)	S(1)	S(1)	S(1)

S= sensible; I= intermediate sensibility; R= resistant

Susceptibilities values (μg/mL) were analyzed according to the Clinical and Laboratory Standards Institute CLSI (2015): CIP (Ciprofloxacin): R=≤15; 1=16-20; S=≥21; IPM (Imipenem): R=≤13; 1=14-15; S=≥16; TET (Tetracycline): R=≤14; 1=15-18; S=≥19; CTX (Cefotaxime): R=≤14; 1=15-22; S=≥23; DOX (Doxycycline): R=≤12; 1=13-15; S=≥16; AMC (amoxicillin + clavulanic acid): R=≤13; 1=14-17; S=≥18; TOB (Tobramycin): R=≤12; 1=13-14; S=≥15; AMO (Amoxicillin): R=≤13; 1=14-16; S=≥17

Moreover, 37.5% and 12.5% of *E. coli* strains were resistant to doxycycline and tobramycin (TM), respectively, while 12.5% of *Enterobacter aerogenes* were resistant to the same antibiotics. In addition, 25% of species of *Citrobacter freundii* were resistant to doxycycline and imipenem (IP) and 33.3% of *E. cloacae* demonstrated insensitivity to doxycycline. The *Serratia marcescens* strains showed resistance to cefotaxime and to tobramycin. The two (100%) *Serratia liquefaciens* strains and the only one *Pseudomonas aeruginosa* (50%) showed values of MIC_90_ for doxycycline and cefotaxime greater than 16 μg/mL and 64 μg/mIL, respectively (Tables 6 and 7).

**Table 6:** Percentage of susceptibility of multiresistant microbial species isolated from dental prostheses biofilm regarding Minimum Inhibitory Concentration (MIC) of five antibiotics tested

Microorganism(n)	CT*	TM*	DC*	IP*	CI*
***Klebsiella pneumoniae* (n=9)**					
Sensible	66.6	88.9	77.8	77.8	79.8
Intermediate sensibility	0.0	0.0	0.0%	11.1	11.1
Resistent	33.4	11.1	22.2	11.1	11.1
***Escherichia coli* (n=8)**					
Sensible	87.5	87.5	37.5	100.0	100.0
Intermediate sensibility	12.5	0.0	25.0	0.0	0.0
Resistent	0.0	12.5	37.5	0.0	0.0
***Enterobacter aerogenes* (n=8)**					
Sensible	100.0	87.5	75.0	100.0	100.0
Intermediate sensibility	0.0	0.0	15.5	0.0	0.0
Resistent	0.0	12.5	12.5	0.0	0.0
***Citrobacter freundii* (n=4)**					
Sensible	100.0	100.0	75.0	75.0	100.0
Intermediate sensibility	0.0	0.0	0.0	0.0	0.0
Resistent	0.0	0.0	25.0	25.0	0.0
***Serratia marcescens* (n=1)**					
Sensible	0.0	0.0	100.0	100.0	100.0
Intermediate sensibility	0.0	0.0	0.0	0.0	0.0
Resistent	100.0	100.0	0.0	0.0	0.0
***Enterobacter cloacae* (n=3)**					
Sensible	100.0	100.0	66.7	100.0	100.0
Intermediate sensibility	0.0	0.0	0.0	0.0	0.0
Resistent	0.0	0.0	33.3	0.0	0.0
***Serratia liquefaciens* (n=2)**					
Sensible	100.0	100.0	0.0	50.0	100.0
Intermediate sensibility	0.0	0.0	0.0	0.0	0.0
Resistent	0.0	0.0	100.0	50.0	0.0
***Enterobacter gergoviae* (n=1)**					
Sensible	0.0	100.0	0.0	100.0	100.0
Intermediate sensibility	0.0	0.0	100.0	0.0	0.0
Resistent	100.0	0.0	0.0	0.0	0.0
***Pseudomonas aeruginosa* (n=2)**					
Sensible	50.0	100.0	50.0	100.0	100.0
Intermediate sensibility	0.0	0.0	50.0	0.0	0.0
Resistent	50.0	0.0	0.0	0.0	0.0
***Pseudomonas putida* (n=1)**					
Sensible	100.0	100.0	100.0	100.0	100.0
Intermediate sensibility	0.0	0.0	0.0	0.0	0.0
Resistent	0.0	0.0	0.0	0.0	0.0
***Burkholderia pseudomallei* (n=1)**					
Sensible	100.0	100.0	100.0	100.0	100.0
Intermediate sensibility	0.0	0.0	0.0	0.0	0.0
Resistent	0.0	0.0	0.0	0.0	0.0

* CT (0.002-32 μg/mL), TM (0.016-256 μg/mL), DC (0.016-256 μg/mL), IP (0.002-32 μg/mL), CI (0.002-32 μg/mL); Susceptibilities values (μg/mL) were analyzed according to the Clinical and Laboratory Standards Institute CLSI (2015): Cefotaxime (CT) S≤8, 16≤l≤32, R≥64; Tobramycin (TM) S≤4, l=8, R≥16; Doxicyclin (DC) S≤4, l=8, R≥16; Imipenem (IP) S≤4, l=8, R≥16; Ciprofloxacin (CI) S≤1, l=2, R≥4

**Table 7:** Minimum Inhibitory Concentration (MIC) values of five antibiotics* tested for multiresistant microbial species isolated from dental prostheses biofilm

Microorganism	CT**	TM**	DC**	IP**	CI**
*Klebsiella pneumoniae*	>32	12	0.19	0.75	0.25
*Klebsiella pneumoniae*	0.094	2	2	>32	0.047
*Klebsiella pneumoniae*	0.5	2	2	0.38	0.032
*Klebsiella pneumoniae*	0.047	3	2	0.25	0.064
*Klebsiella pneumoniae*	0.064	3	2	0.25	0.064
*Klebsiella pneumoniae*	0.032	3	48	0.19	0.047
*Klebsiella pneumoniae*	>32	3	16	6	3
*Klebsiella pneumoniae*	>32	3	2	0.25	12
*Klebsiella pneumoniae*	0.032	3	2	0.25	0.064
*Escherichia coli*	0.38	16	6	0.38	0.064
*Escherichia coli*	0.25	1.5	3	0.38	0.064
*Escherichia coli*	0.125	3	12	0.25	0.023
*Escherichia coli*	12	0.75	32	1.5	0.125
*Escherichia coli*	0.047	2	1.5	0.125	0.023
*Escherichia coli*	0.064	3	6	0.19	0.094
*Escherichia coli*	0.125	2	0.5	0.125	0.016
*Escherichia coli*	2	2	12	0.19	0.047
*Enterobacter aerogenes*	0.25	24	6	0.38	1
*Enterobacter aerogenes*	0.25	2	3	0.38	0.064
*Enterobacter aerogenes*	0.032	3	3	0.25	0.047
*Enterobacter aerogenes*	0.25	3	>256	0.38	0.064
*Enterobacter aerogenes*	0.047	2	2	0.19	0.047
*Enterobacter aerogenes*	0.016	0.75	1.5	3	0.064
*Enterobacter aerogenes*	8	2	2	0.25	0.064
*Enterobacter aerogenes*	0.19	3	2	0.25	0.047
*Citrobacter freundii*	0.25	3	>256.0	0.25	0.032
*Citrobacter freundii*	0.25	3	3	>32	0.047
*Citrobacter freundii*	0.094	2	3	0.25	0.047
*Citrobacter freundii*	0.25	3	3	0.25	0.047
*Serratia marcescens*	3	64	0.5	0.5	0.75
*Enterobacter cloacae*	0.064	0.75	3	0.19	0.012
*Enterobacter cloacae*	3	0.75	24	0.19	0.032
*Enterobacter cloacae*	0.38	2	3	0.38	0.023
*Serratia liquefaciens*	3	0.5	16	0.38	0.047
*Serratia liquefaciens*	8	2	>256	>32	0.5
*Enterobacter gergoviae*	>32	1	6	0.5	0.047
*Pseudomonas aeruginosa*	>32	1	8	0.38	0.064
*Pseudomonas aeruginosa*	8	1	1.5	1.5	0.047
*Pseudomonas putida*	0.5	0.023	0.25	0.032	0.047
*Burkholderia pseudomallei*	2	0.5	3	0.19	0.003
*Burkholderia pseudomallei (n=1)*					

* CT (0.002-32 μg/mL), TM (0.016-256 μg/mL), DC (0.016-256 μg/mL), IP (0.002-32 μg/mL), CI (0.002-32 μg/mL)

** μg/mL

## DISCUSSION

Enteric bacilli and pseudomonads are opportunistic pathogens at different human body sites. The oral cavity functions as a reservoir for pathogenic species^3,17^. As a result of the aging process, the oral health status declines, often leading to defective oral/denture hygiene in geriatric subjects, being the reasons for this fact the lack of hygiene education of these subjects or of those caring for them. Consequently, oral hygiene is often neglected, resulting in poor oral health and in an increase in the presence of local or general infections that can be related with the presence of enteric microorganisms in the oral cavity^4,9^.

Here we show that dental prostheses can harbor opportunistic pathogens, such as those identified in this study; in addition, these microorganisms can be even resistant to antimicrobial drug therapy as demonstrated here. This is really alarming because, albeit many of these subjects might not be systemically compromised by any disease at the moment they house such pathogens in their oral cavity, they could suffer from any illness that could dampen and compromise their immune system, making them, in turn, more susceptible to be infected by the oral opportunistic pathogens they are harboring.

Thus, a proper denture hygiene protocol is essential, since it could prevent these individuals from exposition to these bacterial species, considering that respiratory pathogens are more capable of colonizing teeth and dentures instead of soft tissues, and pneumonia is the main cause of death related to infection in older people^8^. By wearing dental prostheses, individuals are at a higher risk of aspirating such pathogens from the denture biofilm due to the close proximity of this oral appliance and lungs, being reported a high prevalence of respiratory pathogens on the denture biofilm of hospitalized adults^21^. Furthermore, not only are such pathogens involved in the development of aspiration pneumonia, but also they have been directly associated with bacterial endocarditis, gastrointestinal infection, and chronic obstructive pulmonary disease. This scenario shows us that daily removal of the denture biofilm is important to prevent the occurrence of associated oral and systemic diseases^10^.

Regarding the prevalence of the aforementioned microorganisms, we found a high prevalence (65.4%) of *Enterobacteriaceae* and *Pseudomonadaceae* species in the denture biofilm from individuals living in the city of Sobral. As opposed to a similar study in a Japanese population, in which the prevalence of potential pathogens on dentures was 18% for *E. cloacae* and 16% for *K. pneumoniae,* our research found that *K. pneumoniae* was the most prevalent at 26.5%, followed by *Escherichia coli* and *E. aerogenes,* placed after 23.5%^28^. Another study showed that denture plaque in patients with chronic obstructive pulmonary disease was colonized by pathogens of the respiratory tract, including: *E. coli, Pseudomonas spp.,* and *Klebsiella spp.* Over 33% of the isolated pathogens are part of rod-shaped and gram-negative *Enterobacter spp.* The isolation of the mentioned bacteria from denture plaque proves that dental prostheses might become a source of infection of the respiratory tract or may exacerbate chronic respiratory diseases^25^.

Contrary to the data we report, another group described a much lower (20.3%) prevalence of enterobacteriaceae in the oral cavity of older people from Greece that used dental prostheses^14^, whereas a different group reported that 48% of edentulous subjects harbored enteric rods in their oral cavity, being *K. oxytoca, E. cloacae,* and *K. pneumoniae* species more prevalent^12^. According to these authors, the prevalence of such microorganisms in the denture biofilm may be related to the ability of these species to adhere to the polymer of the denture and to aggregate in the presence of ammonium sulfate. This could explain the high prevalence of *K. pneumoniae* reported in our study.

The classic literature shows that the prevalence of these organisms in the oral cavity of systemically healthy individuals can vary among different populations^1,26,27^. Thus, the high prevalence (65.4%) we observed in this study could be attributed to disadvantaged health infrastructure and educational issues that could have led to ingestion of contaminated food or water. Also, poor hygiene habits and indiscriminate use of antibiotics may play a role in this high prevalence^4,11^.

In fact, it was observed that the prostheses hygiene is often precarious. Among the difficulties encountered when conducting a properly cleaning, we can include the lack of adequate patient guidance, characteristics of the prostheses, decreased motor ability, and lack of proper products in the market to carry out the correct denture cleaning. The habit of brushing the prostheses with toothpaste and the use of common brushes may not be the best way to achieve efficient cleaning. Another factor is that the prolonged use of the same prostheses for many years might contribute to the colonization process by potential pathogens^4^. It is relevant to highlight that this information shows a major limitation of these studies, since there are many factors that can lead to microbial colonization of dentures. In our study, we found prostheses being worn for 40 years, and most of the subjects wearing contaminated dentures have been doing so for over 10 years.

Regarding the antimicrobial susceptibility of microorganisms, our data show that 86.5% of anaerobic facultative gram-negative bacilli were resistant to amoxicillin and over half of them had resistance to amoxicillin with clavulanic acid contrary to other studies that showed the association amoxicillin/clavulanic acid being active on less than half of ampicillin or amoxicillin resistant isolates from prostheses biofilm. Resistance to this association was detected in 28.3% of the targeted microorganisms and it was particularly frequent in *E. cloacae, genera Klebsiella, Serratia,* and *Pseudomonas* ^11^.

The analysis of the MIC proved that the antimicrobial drug with the highest inhibitory activity against the enteric bacilli and pseudomonads was ciprofloxacin (MIC_90_≤1 μg/mL). Nonetheless, we observed a decreased susceptibility to this antimicrobial agent in two (22.2%) strains of *K. pneumoniae* showing MIC values of 3 and 12 μg/mL, respectively. Although it is a much lower frequency than that observed previously^20^, decreased susceptibility rates to ciprofloxacin (MIC_90_>2 μg/ mL) among clinical isolates of *Enterobacteriaceae* species is a particular concern, since enteric rods were able to colonize the denture surface of non-hospitalized subjects, which could spread fluoroquinolones-resistant enterobacteria clones in the community. Fluoroquinolones are the most widely used antibiotics worldwide, and are the drugs of choice for empirical therapy for urinary tract infection. Fluoroquinolone resistance in members of the *Enterobacteriaceae* family has until recently been attributed to mutations in the gyrase and topoisomerase genes quinolone resistance-determining regions. Given their transferability and the possibility that they cause increases in resistance that might affect the clinical response to treatment, the detection of quinolone resistance should be routinely performed. There are several surveillance or retrospective studies with clinical isolates, and these studies showed the characteristic features observed in drug-resistant strains in addition to epidemics caused by them^33^.

In this study we also found a slightly higher prevalence (27.5%) of *Enterobacteriaceae* species with decreased susceptibility to cefotaxime (MIC_90_≥2 μg/mL) than the data previously reported of 22.5%^21^, and these isolates were considered potential ESBL (Extended Spectrum Beta-Lactamases) producers according to the limits established by the CLSI^6,7^. Resistance to β-lactam antimicrobials in *Enterobacteriaceae* has been largely due to the presence of β-lactamase enzymes. β-lactamase genes were originally found to be chromosomal. β-lactamase-mediated resistance may develop *in vivo* during chemotherapy, lending support to the prevalent view that ESBL plasmids are conjugative, may be borne on transposons, and that the genes may have high mutation frequencies. Treatment of infections caused by these ESBL-producing bacteria has become challenging. In addition to being resistant to commonly used extended spectrum β-lactams, these isolates are usually resistant to other classes of antibiotics including fluoroquinolones and aminoglycosides at the same time. One reason for such resistance profile is the practice of irrational usage of antibiotics, leading these microorganisms to exhibit a unique microorganism resistant pattern, which hugely impacts on clinical choice of a correct antibiotic once antimicrobial drug resistance develops, making difficult to treat systemic infections^15,22,23^.

Antimicrobial resistance surveillance programs have provided sufficient data about antimicrobial susceptibility of clinically relevant enteric bacteria and pseudomonads from nosocomial infections and the environment^5,18^. However, they can be found in the biofilm formed on dentures, increasing the risk of the individuals to systemic infections or even worsening them. Thus, it is crucial to emphasize that dentures can harbor such pathogens and this is the reason why a correct hygiene protocol must be carried out in order to avoid such biofilm accumulation on dental prostheses, which, ultimately, would increase the risk of one to be exposed to enteric bacilli.

## CONCLUSION

*K. pneumoniae, E. coli,* and *E. aerogenes* were the predominant species found on the denture biofilm. Most enteric bacilli and *Pseudomonas* spp. were resistant to amoxicillin and amoxicillin clavulanate, with variable susceptibility patterns to other antimicrobial drugs. The antibiotic that showed the highest inhibitory activity against them was ciprofloxacin, but we also found the emergence of ciprofloxacin-reduced sensitivity strains. Therefore, from the results obtained in this study, we suggest that preventative programs for the biofilm control is important to avoid the colonization of dental prostheses by multidrug resistant bacteria as well as avoiding the indiscriminate prescription of antibiotics will help diminish the multidrug resistance insurgence.
